# The Medical and Surgical Report

**Published:** 1904-08

**Authors:** 


THE MEDICAL AND SURGICAL REPORT of the Presbyterian Hospital in the city of New York is just out and it contains some very able papers, together with a number of fine illustrations. Dr. John Howland's paper on the "Pathological Anatomy of Shiga Bacillus Infection of the Intestines in Infants," won the Briddon medal. The paper is very interesting and instructive.

